# Computed Tomography–Based Evaluation of Airway Remodeling for Distinguishing Asthma–Chronic Obstructive Pulmonary Disease Overlap From Asthma and Chronic Obstructive Pulmonary Disease

**DOI:** 10.1155/carj/4348540

**Published:** 2026-05-30

**Authors:** Mei-Cheng Chen, Yang-Li Liu, Feng-Jia Chen, Wei Liang, Wei-Wei Deng, Ling Ma, Yu-Biao Guo, Shi-Ting Feng, Ying Zhu

**Affiliations:** ^1^ Department of Radiology, The First Affiliated Hospital of Sun Yat-Sen University, Guangzhou, 510080, Guangdong, China, sysu.edu.cn; ^2^ Division of Pulmonary and Critical Care Medicine, The First Affiliated Hospital of Sun Yat-Sen University, Guangzhou, 510080, Guangdong, China, sysu.edu.cn; ^3^ Department of Medical Records Management, The First Affiliated Hospital of Sun Yat-Sen University, Guangzhou, 510080, Guangdong, China, sysu.edu.cn; ^4^ Clinical & Technical Support, Philips Healthcare, Shanghai, 200072, Shanghai, China, philips.com

**Keywords:** asthma–COPD overlap syndrome (ACO), diagnostic model, lung function, X-ray computed tomography

## Abstract

**Objectives:**

We aimed to explore the practicality of CT imaging for diagnosing asthma–chronic obstructive pulmonary disease (COPD) overlap syndrome (ACO).

**Method:**

A total of 339 patients, including asthma, COPD, and ACO, were retrospectively collected from our hospital from February 2014 to October 2020. Airway and emphysema CT parameters were measured by image‐analyzing software. Multivariate logistic regression analysis was applied to select independent variables for building the diagnostic models.

**Result:**

Female gender, younger age, higher WA^1st^/BSA (the mean wall area of the trachea and main bronchi), and lower WT^1st^/BSA (the mean wall thickness of the trachea and main bronchi) were independently associated with a higher risk of developing ACO compared to COPD. Male gender, older age, lower inferior lobes mean density (MD), higher inferior lobes WA^2nd^% (the mean wall area of the segmental bronchus), and lower superior lobes WA^2nd^% were independently associated with a higher risk of developing ACO compared to asthma. The area under the receiver operating characteristic curves (AUCs) of the two differential diagnostic models for ACO was 0.736 and 0.826, respectively. The calibration curve represented good consistency between the predictions by the models and the actual observations.

**Conclusion:**

The proposed models can serve as noninvasive tools to diagnose ACO from COPD and asthma. This study highlights the value of CT in evaluating airway remodeling features and holds promise for clinical application.

## 1. Introduction

Despite asthma and chronic obstructive pulmonary disease (COPD) having distinct pathophysiological mechanisms, a noticeable portion of patients have overlapping clinical manifestations [1], which is termed asthma–COPD overlap syndrome (ACO). Recent evidence warrants attention that, compared with patients with asthma or COPD alone, patients with ACO have poor respiratory symptom control, more frequent exacerbations, a higher rate of hospitalization [2], and more severe lung function [3, 4]. The variations in mortality rates associated with ACO may arise from discrepancies in its definition across studies or from differences in therapeutic approaches, notably regarding the use of inhaled corticosteroids (ICS). While pivotal in asthma, their varied application in ACO patients underscores the criticality of identifying ACO in clinical practice, particularly in discerning individuals who may benefit from early initiation of ICS [5, 6]. Moreover, diagnosing ACO in COPD patients is significant, given their heightened responsiveness to ICS [7, 8]. ACO patients may find merit in combined therapy encompassing a triple regimen comprising β2‐agonists, anticholinergic bronchodilators, and ICS [9]. Additionally, personalized education and self‐management plans for mixed conditions must cater to individualized needs, reflecting unique objectives and treatment expectations distinct from those with singular diseases [10, 11].

To date, most of the criteria of ACO are in view of clinical symptoms and the values of lung function tests [12], such as FEV1, FEV1/FVC, and the bronchodilator test. However, the lung function test has limitations. First, the FEV1:FVC ratio is not sufficiently sensitive for mild airway disease due to the strong compensatory ability of the lung [13, 14]. In addition, many hospitals in rural and remote regions lack spirometer services [15]. Moreover, in the current healthcare context, spirometry tests are sometimes restricted due to concerns about the potential for aerosol transmission, such as during the COVID‐19 pandemic [16]. Since the inadequate use of spirometers leads to an inadequate diagnosis of the disease, the criteria proposed are challenging to use in real practice. It is crucial to propose safe and sensitive diagnosis criteria for ACO to reduce the worldwide disease burden.

Nowadays, technical advancement in computed tomography scans offers the capacity to explore the early structural abnormalities of the airway [14, 17, 18]. Gao et al. [19] stated that CT densitometry presents both quantitative lung density data and volumetric information. ACO patients have a lower degree of emphysema and greater postbronchodilator variations in air trapping than COPD patients. Niwa et al. [20] have stated that patients with ACO had a thicker airway wall than asthma patients at the level of the segmental bronchi. Xie et al. [21] found that asthma patients with a high emphysema index (EI) have the features of ACO and that quantitative CT measurements of emphysema may help diagnose ACO. However, previously published studies have yet to plumb the depths of the structural changes among asthma, COPD, and ACO in the airway and develop individualized diagnosis models [22].

Since CT imaging can offer a different perspective from the capabilities of traditional functional tests and play as a supplementary diagnostic tool in chronic airway disease evaluation, by providing a more comprehensive understanding of both anatomical and functional dimensions, as well as the morphology change in airways, we hypothesized that the airway remodeling features between asthma, COPD, and ACO could be found via CT. Specifically, we aimed to explore potentially significant CT parameters associated with ACO among ACO, COPD, and asthma groups. Moreover, we aimed to build up simple CT‐based diagnosis models for ACO in clinical practice and guide the clinical treatment and long‐term management. To our knowledge, these approaches have not been previously reported.

## 2. Methods

### 2.1. Subject

Three study groups including 123 COPD patients, 108 asthma patients, and 108 ACO patients were retrospectively collected in the First Affiliated Hospital of Sun Yat‐sen University from February 2014 to October 2020. This study was approved by the ethics committee of our institution with a waiver of informed consent (No. [2021]072). The diagnosis of asthma and COPD was according to the GINA and GOLD definitions [23, 24], respectively. In this study, the diagnosis of ACO was made when patients met either one of two criteria [25]: (1) in asthmatic patients, older than 40 years old and had a smoking history of ≥ 10 pack‐years, air pollution exposure (from wood or coal; exposure ≥ 100 h/year), and FEV1/FVC < 0.7, and (2) in COPD patients, history of asthma before the age of 40, positive bronchodilator test defined as an increase in FEV1 > 200 mL and > 12%. Control subjects were recruited from patients with normal lung function (FEV_1_ ≥ 80% predicted).

Criteria for excluding the subjects were as follows: (1) patients with other chronic lung diseases such as lung cancer and bronchiectasis; (2) patients who had undergone lung resection; (3) patients whose results could not be corrected due to segmentation errors by postprocessing software; and (4) patients with incomplete clinical data.

### 2.2. Study Design and Endpoint

**FIGURE 1:**
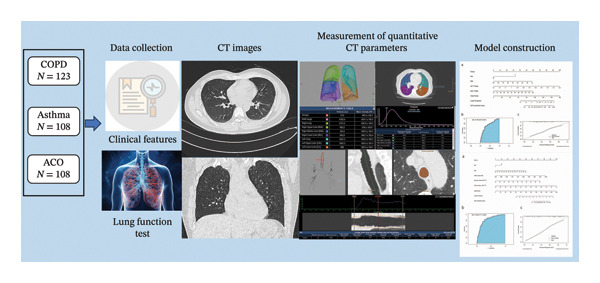
Flowchart of the study.

First, the study recruited patients in three groups, including ACO, COPD, and asthma groups. The independent clinical and CT characteristics were identified. Based on the results of our preliminary experiment, a sample size calculation was performed in R using a two‐sample *t*‐test for CT parameters. With a two‐sided *α* of 0.05, power of 0.80, and 1:1 group allocation, the calculation indicated that a minimum of 100 cases per group was sufficient to detect significant differences in CT parameters between the two groups. Second, individualized models for differentiating ACO from the COPD and asthma groups were built and internally validated, respectively (Figure 1).

### 2.3. Thin‐Section CT and Measurement of Quantitative CT Parameters

All participants underwent imaging with IQon Spectral CT (Philips Healthcare, Best, The Netherlands) or SOMATOM Definition AS+ (Siemens Healthineers, Forchheim, Germany) at full inspiration in the supine position. Scan parameters were as follows: detector collimation width of 64 × 0.6 mm or 128 × 0.6 mm; tube voltage of 120 kV; and tube current of 40–80 mA, regulated by the automatic exposure control system. Slice thicknesses were set at 1.25 mm or 2.00 mm and intervals were set at 1.25 mm or 2.00 mm. Slice thickness of 2.00 mm was used in 11/123 (8.9%) patients in the COPD group, 10/108 (9.2%) in the asthma group, and 11/108 (10.1%) in the ACO group. All images were reconstructed with lung and soft tissue kernels for quantitative analysis, including a B70f reconstruction kernel and a mediastinal B30f kernel.

Quantitative CT parameters were measured by using image analysis software (Philips intelliSpace Portal). We used an integral‐based method for airway measurements [26, 27]. Lung density parameters, including mean density (MD) of the lung, emphysema ratio (ER) of the lung, and bronchial parameters of first‐ and third‐generation airways, including wall thickness (WT, mm), wall area (WA, sq. mm), and wall area percentage (WA%) (Supporting Information Figure S1), were measured. To simplify the calculation, we defined the trachea and main bronchus as the first‐level airway and defined the lobar bronchus as the second‐level airway. Detailed procedures are described in the Supporting Information.

### 2.4. Measurement of Lung Function Tests

The following lung function parameters were acquired: the FEV1, FEV1% predicted, and FVC. Detailed procedures are described in the Supporting Information.

### 2.5. Statistical Analysis

All statistical analyses were performed using R software (Version 4.1.2, from https://www.r-project.org). All numeric data were expressed as means or medians depending upon normal or nonnormal distribution, respectively. Categorical variables were expressed as numbers (%). The difference between groups was examined with a *t*‐test, Kruskal–Wallis test, and chi‐square test. Parameters from univariate analyses with a *p* < 0.1 were selected for the multivariate logistic regression analyses. Finally, the area under the receiver operating characteristic curve (AUC) was calculated to evaluate the diagnostic value of the multivariate models and assessed by the sensitivity, specificity, positive predictive value, and negative predictive value. Diagnostic models were formulated based on the results of multivariate logistic regression analyses. The predictive performance of the established models was assessed by a calibration curve. *p* < 0.05 values were considered statistically significant, and all tests are two‐sided.

## 3. Results

### 3.1. Subject Characteristics

Patient clinical characteristics and CT parameters of ACO and non‐ACO groups, including COPD and asthma, are shown in Table 1. When compared with the non‐ACO group, significant differences in sex, body mass index (BMI), and age were found between the ACO and non‐ACO groups. Patients with ACO were younger than those in the COPD group and older than those in the asthma group. The female ratio and BMI were significantly higher in patients with ACO than in COPD patients but lower than in asthma patients. The pulmonary function values, including FEV1% predicted, FVC% predicted, and the FEV1:FVC ratio of the ACO group, tended to be significantly higher than those in the COPD group and significantly lower than those in the asthma group.

**TABLE 1:** Characteristics of the subjects.

	[All] *N* = 339	ACO *N* = 108	Asthma *N* = 108	COPD *N* = 123	p.overall	p.ACO vs. asthma	p.ACO vs. COPD	p.asthma vs. COPD
Sex					< 0.001	0.006	< 0.001	< 0.001
F	102 (30.1%)	34 (31.5%)	55 (50.9%)	13 (10.6%)				
M	237 (69.9%)	74 (68.5%)	53 (49.1%)	110 (89.4%)				
BMI (kg/m^2^)	22.1 [17.3; 25.2]	22.2 [18.4; 25.9]	23.5 [20.7; 26.2]	19.6 [15.2; 24.4]	< 0.001	0.078	0.003	< 0.001
BSA (m^2^)	1.64 [1.52; 1.75]	1.67 [1.52; 1.80]	1.67 [1.53; 1.79]	1.62 [1.52; 1.71]	0.052	0.721	0.112	0.062
Age (years)	64.0 [54.0; 71.0]	63.5 [52.8; 71.0]	56.0 [47.0; 64.2]	70.0 [63.5; 75.0]	< 0.001	< 0.001	< 0.001	< 0.001
BothLungs_ER	9.30 [2.40; 22.0]	10.7 [2.28; 20.8]	5.75 [0.95; 20.7]	11.0 [3.40; 24.4]	0.019	0.065	0.46	0.021
Superior lobes ER	11.1 [3.03; 22.9]	12.9 [3.05; 22.6]	7.42 [1.67; 22.3]	12.3 [3.68; 24.1]	0.115	0.156	0.734	0.156
Inferior lobes ER	7.35 [1.58; 19.2]	9.35 [1.61; 17.2]	4.05 [0.35; 16.7]	8.30 [2.60; 22.3]	0.008	0.048	0.521	0.006
Both lungs_MD (HU)	−859.40 [‐882.55;‐826.00]	−861.80 [‐885.55;‐835.65]	−829.00 [‐864.03;‐801.25]	−870.40 [‐891.15;‐848.75]	< 0.001	< 0.001	0.048	< 0.001
Superior lobes MD	−867.37 [‐888.25;‐838.12]	−868.22 [‐892.24;‐844.77]	−845.48 [‐873.89;‐821.93]	−878.20 [‐896.47;‐857.10]	< 0.001	< 0.001	0.063	< 0.001
Inferior lobes MD	−845.45 [‐875.95;‐808.25]	−847.58 [‐875.92;‐820.64]	−815.50 [‐855.56;‐777.74]	−858.65 [‐881.80;‐826.92]	< 0.001	< 0.001	0.113	< 0.001
WT^1st^/BSA (mm/m^2^)	0.82 [0.74; 0.91]	0.82[0.73; 0.92]	0.79 [0.71; 0.87]	0.86 [0.78; 0.94]	< 0.001	0.01	0.032	< 0.001
WA^1st^/BSA (mm^2^/m^2^)	43.5 [37.1; 49.2]	43.7 [38.7; 49.3]	37.4 [32.9; 44.0]	46.0 [42.0; 51.4]	< 0.001	< 0.001	0.052	< 0.001
WA^1st^%	28.1 [25.7; 30.9]	27.9 [25.8; 30.9]	28.8 [25.9; 31.2]	27.9 [25.4; 30.8]	0.39	0.509	0.696	0.509
Superior lobes WT^2nd^/BSA	0.87 [0.77; 0.97]	0.89 [0.76; 0.98]	0.83 [0.76; 0.96]	0.87 [0.79; 0.98]	0.355	0.414	0.958	0.414
Superior lobes WA^2nd^/BSA	27.7 (5.42)	28.6 (5.32)	26.2 (5.43)	28.2 (5.27)	0.002	0.004	0.858	0.014
Superior lobes WA^2nd^%	42.6 (7.42)	41.6 (7.98)	44.7 (7.26)	41.5 (6.65)	0.001	0.005	0.993	0.003
Inferior lobes WT^2nd^/BSA	0.89 [0.80; 1.01]	0.92 [0.83; 1.01]	0.82 [0.74; 0.94]	0.92 [0.84; 1.05]	< 0.001	< 0.001	0.658	< 0.001
Inferior lobes WA^2nd^/BSA	28.2 [23.8; 31.3]	28.8 [24.8; 31.5]	26.5 [22.6; 30.0]	28.7 [25.5; 31.9]	0.001	0.003	0.92	0.002
Inferior lobes WA^2nd^%	44.4 [39.7; 50.8]	45.8 [40.9; 50.3]	42.4 [37.9; 48.5]	44.9 [40.4; 52.1]	0.01	0.015	0.781	0.015
FVC% predicted	73.3 (20.5)	69.4 (19.6)	87.7 (17.5)	64.0 (16.6)	< 0.001	< 0.001	0.058	< 0.001
FEV1% predicted	52.8 (24.6)	44.2 (16.9)	79.0 (19.2)	37.4 (14.0)	< 0.001	< 0.001	0.001	< 0.001
FEV1/FVC	54.0 [42.5; 68.0]	51.0 [41.8; 58.2]	74.0 [68.0; 79.2]	44.0 [38.5; 53.0]	< 0.001	< 0.001	0.002	< 0.001

*Note:* Values are shown as the number (percentage), mean (SD), or median [interquartile range]. ACO, asthma–chronic obstructive pulmonary disease overlap; emphysema ratio, the % area of low attenuate (<‐950HU) to the corresponding lung area; superior lobes, upper lobes of both lungs and middle lobe of right lung; inferior lobes, lower lobes of both lungs; WT, the wall thickness of airways; WA, the wall area of airways; WA%, wall area percentage; WT^1st^/BSA, first‐level airway wall thickness divided by body surface area. FEV1, forced expiratory volume in 1 s.

Abbreviations: BMI, body mass index; BSA, body surface area; COPD, chronic obstructive pulmonary disease; FVC, forced vital capacity.

### 3.2. CT Parameters in the ACO, Asthma, and COPD Groups

**FIGURE 2:**
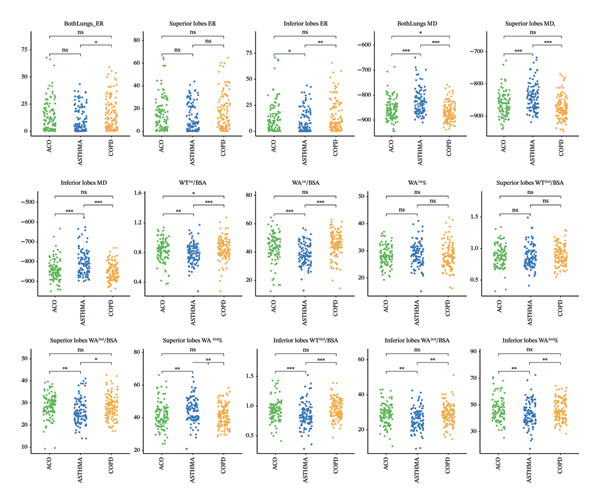
Comparisons of quantitative CT parameters among ACO, COPD, and ASTHMA groups. Note: ^∗^ *p* < 0.05; ^∗∗^ *p* < 0.01; ^∗∗∗^ *p* < 0.001. Abbreviations: ACO, asthma–COPD overlap; COPD, chronic obstructive pulmonary disease; BSA, body surface area; superior lobes, the upper lobe of both lungs and middle lobe of the right lung; inferior lobes, lower lobes of both lungs; MD, mean density; ER, emphysema ratio; WT, the wall thickness of airway; WA, wall area of the airway; WA%, wall area percentage; WT^1st^/BSA, the mean wall thickness of the trachea and main bronchus divided by body surface area; WT^2nd^/BSA, the mean wall area of the lobar bronchus divided by body surface area.

The results of CT parameters are presented as the mean (SD) or medians (interquartile ranges) in Table 1. Compared with the asthma groups, both lungs’ ER and the inferior lobes ER were significantly higher in patients with COPD. There was no significant difference in the superior lobes ER among the three groups (Figure 2).

Both lungs’ MD in the ACO group tended to be significantly lower than that in the asthma group and higher than that in the COPD group. The superior lobes MD and inferior lobes MD were significantly different between the asthma groups and the other two groups, while the values in the COPD group were the highest among the three groups. No significant difference in the superior lobes MD and inferior lobes MD was observed between the ACO and COPD groups.

Regarding the CT parameters of the airways, the first‐level airway WT/BSA (WT^1st^/BSA) was significantly different in every two groups, while the value in the ACO group was higher than that in the asthma group and lower than that in the COPD group. The first‐level airway WA/BSA (WA^1st^/BSA), superior lobes bronchus WA/BSA (superior lobes WA^2nd^/BSA), inferior lobes bronchus WT/BSA (inferior lobes WT^2nd^/BSA), inferior lobes bronchus WA/BSA (inferior lobes WA^2nd^/BSA), and inferior lobes bronchus WA/airway area (inferior lobes WA^2nd^%) of the patients with asthma tended to be significantly lowest among the three groups. Patients with asthma had a higher superior lobes bronchus WA/airway area (superior lobes WA^2nd^%) than patients with ACO and COPD. There was no significant difference in the first‐level airway WA/airway area (WA^1st^%) and superior lobes bronchus WT/BSA (superior lobes WT^2nd^/BSA) among the three groups.

### 3.3. Establishment of CT Model to Differentiate ACO From COPD and Its Diagnostic Efficacy

The parameters sex, age, BMI, BSA, both lungs MD, superior lobes MD, inferior lobes MD, WA^1st^/BSA, and WT^1st^/BSA were included in the multivariate analysis, and the result is presented in Table 2. The sex, age, WA^1st^/BSA, and WT^1st^/BSA were the independent risk factors of ACO (sex OR: 0.09, 95% CI: 0.03–0.24, *p* < 0.001; age OR: 0.95, 95% CI: 0.92–0.97, *p* < 0.001; WA^1st^/BSA OR: 1.08, 95% CI: 1.01–1.16, *p* = 0.029; and WT^1st^/BSA OR: 0.00, 95% CI: 0.00–0.12, *p* = 0.004). A diagnostic nomogram was built for the differential diagnosis of ACO from COPD based on the independent risk factors confirmed by multivariable logistic regression analysis (Table 2, Figure 3(a)). The receiver operating characteristic (ROC) analysis indicated that AUC for diagnosing ACO was 0.736 (95% CI, 0.672–0.801) (Figure 3(b)), and the results of sensitivity, specificity, positive predictive value, and negative predictive value are presented in Table 3. The calibration curve (chi‐square statistic = 2.34, *p* value > 0.05) showed that the differential diagnostic CT model of ACO from COPD was well calibrated in the internal training cohort (Figure 3(c)).

**TABLE 2:** Univariate and multivariate analysis of CT parameters for differentiating ACO from COPD.

Characteristic	OR (univariate)	OR (multivariate)
Sex		
F		
M	0.26 (0.13–0.52, *p* < 0.001)	0.09 (0.03–0.24, *p* < 0.001)
BMI	1.06 (1.02–1.10, *p* = 0.005)	
BSA	6.41 (1.23–33.50, *p* = 0.028)	
Age	0.96 (0.93–0.98, *p* < 0.001)	0.95 (0.92–0.97, *p* < 0.001)
Both lungs’ MD	1.01 (1.00–1.01, *p* = 0.045)	
Superior lobes MD	1.01 (1.00–1.01, *p* = 0.075)	
Inferior lobes MD	1.00 (1.00–1.01, *p* = 0.093)	
WT^1st^/BSA	0.12 (0.02–0.84, *p* = 0.032)	0.00 (0.00–0.12, *p* = 0.004)
WA^1st^/BSA	0.97 (0.95–1.00, *p* = 0.073)	1.08 (1.01–1.16, *p* = 0.029)

*Note:* Variables were selected from univariate analysis (*p* < 0.1). *p* < 0.05 was considered statistically significant in multivariate analysis. The results are shown as OR (95% CI, *p* value); superior lobes, the upper lobe of both lungs and middle lobe of the right lung; inferior lobes, lower lobes of both lungs; MD, mean density; WT^1st^/BSA, the mean wall thickness of the trachea and main bronchus divided by body surface area; WA^1st^/BSA, the mean wall area of the trachea and main bronchus divided by body surface area.

Abbreviations: ACO, asthma–COPD overlap; CI, confidence interval; OR, odds ratio.

FIGURE 3The nomogram, ROC, and calibration curve for the CT model to differentiate ACO from COPD. (a) The ACO differential model was shown as a nomogram. (b) Receiver operating characteristic plot for the ACO differential model. (c) Calibration curve of the ACO predicted model. Bias‐corrected curve line, calibration curve by 1000 bootstrap sampling to decrease the overfitting bias; black diagonal line, the trend line of the Hosmer–Lemeshow test. Abbreviations: ACO, asthma–COPD overlap; WT^1st^/BSA, the mean wall thickness of the trachea and main bronchus divided by body surface area; WA^1st^/BSA, the mean wall area of the trachea and main bronchus divided by body surface area.

**Figure:**
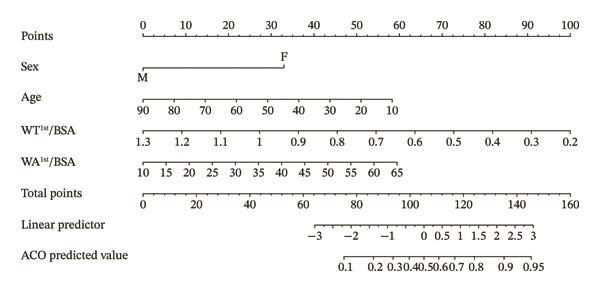
(a)

**Figure:**
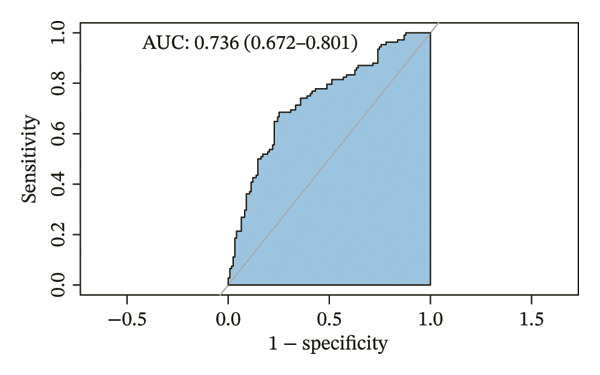
(b)

**Figure:**
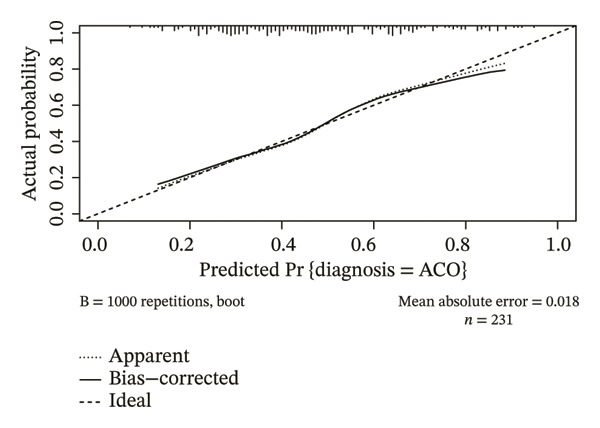
(c)

**TABLE 3:** Receiver operating characteristic analysis for differential diagnostic CT models of ACO.

Model	AUC (95% Cl)	*n*	Sensitivity	Specificity	PPV	NPV
CT model for differentiating ACO from COPD	0.736 (0.672–0.801)	231	0.685	0.748	0.705	0.73
CT model for differentiating ACO from asthma	0.826 (0.771–0.882)	216	0.769	0.787	0.783	0.773

Abbreviations: AUC, area under the curve; NPV, negative predictive value; PPV, positive predictive value.

### 3.4. Establishment of CT Model to Differentiate ACO From Asthma and Its Diagnostic Efficacy

Sex, age, inferior lobes ER, superior lobes MD, inferior lobes MD, WA^1st^/BSA, WT^1st^/BSA, inferior lobes WA^2nd^%, and superior lobes WA^2nd^% were included in the multivariate analysis, and the results showed that sex, age, inferior lobes MD, inferior lobes WA^2nd^%, and superior lobes WA^2nd^% were the independent risk factors of ACO (sex OR: 2.14, 95% CI: 1.05–4.38, *p* = 0.037; age OR: 1.05, 95% CI: 1.03–1.09, *p* < 0.001; inferior lobes MD OR: 0.99, 95% CI: 0.98–0.99, *p* < 0.001; superior lobes WA^2nd^% OR: 0.90, 95% CI: 0.85–0.95, *p* < 0.001; and inferior lobes WA^2nd^% OR: 1.13, 95% CI: 1.08–1.19, *p* < 0.001), as shown in Table 4. A diagnostic nomogram for the differential diagnostic CT model of ACO from asthma was then built (Figure 4(a)). The ROC analysis indicated that the AUC of the model for the diagnosis of ACO from asthma was 0.826 (95% CI, 0.771–0.882) (Figure 4(b)), and the results of sensitivity, specificity, positive predictive value, and negative predictive value are presented in Table 3. The calibration curve (chi‐square statistic = 4.61, *p* value > 0.05) showed that the diagnostic CT model was well calibrated in the internal training cohort (Figure 4(c)).

**TABLE 4:** Univariate and multivariate analysis of CT parameters for differentiating ACO from asthma.

Characteristic	OR (univariate)	OR (multivariate)
Sex		
F		
M	2.26 (1.30–3.93, *p* = 0.004)	2.14 (1.05–4.38, *p* = 0.037)
Age	1.05 (1.02–1.07, *p* < 0.001)	1.05 (1.03–1.09, *p* < 0.001)
Inferior lobes ER	1.02 (1.00–1.04, *p* = 0.074)	
Superior lobes MD	0.98 (0.98–0.99, *p* < 0.001)	
Inferior lobes MD	0.99 (0.98–0.99, *p* < 0.001)	0.99 (0.98–0.99, *p* < 0.001)
WT^1st^/BSA	8.17 (1.16–57.71, *p* = 0.035)	
WA^1st^/BSA	1.07 (1.04–1.11, *p* < 0.001)	
Superior lobes WA^2nd^%	0.95 (0.91–0.98, *p* = 0.004)	0.90 (0.85–0.95, *p* < 0.001)
Inferior lobes WA^2nd^%	1.04 (1.01–1.08, *p* = 0.009)	1.13 (1.08–1.19, *p* < 0.001)

*Note:* Variables were selected from univariate analysis (*p* < 0.1). *p* < 0.05 was considered statistically significant in multivariate analysis. The results are shown as OR (95% CI, *p* value). Superior lobes, the upper lobe of both lungs and the middle lobe of the right lung; inferior lobes, lower lobes of both lungs; WT^1st^/BSA, the mean wall thickness of the trachea and main bronchus divided by body surface area; WA^1st^/BSA, the mean wall area of the trachea and main bronchus; WA^2nd^%, the mean wall area percentage of the lobar bronchus divided by body surface area.

Abbreviations: ACO, asthma–COPD overlap; CI, confidence interval; ER, emphysema ratio; MD, mean density; OR, odds ratio.

FIGURE 4The nomogram, ROC, and calibration curve for the CT model to differentiate ACO from asthma. (a) The ACO differential model was shown as a nomogram. (b) Receiver operating characteristic plot for the ACO differential model. (c) Calibration curve of the ACO predicted model. Bias‐corrected curve line, calibration curve by 1000 bootstrap sampling to decrease the overfitting bias; black diagonal line, the trend line of the Hosmer–Lemeshow test. Abbreviations: ACO, asthma–COPD overlap; superior lobes, the upper lobe of both lungs and the middle lobe of the right lung; inferior lobes, lower lobes of both lungs; MD, mean density; WA^2nd^%, the mean wall area percentage of the lobar bronchus.

**Figure:**
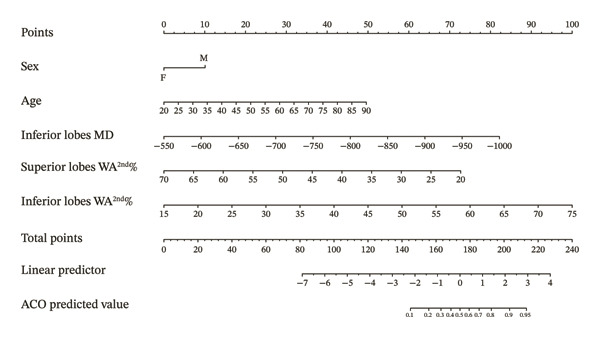
(a)

**Figure:**
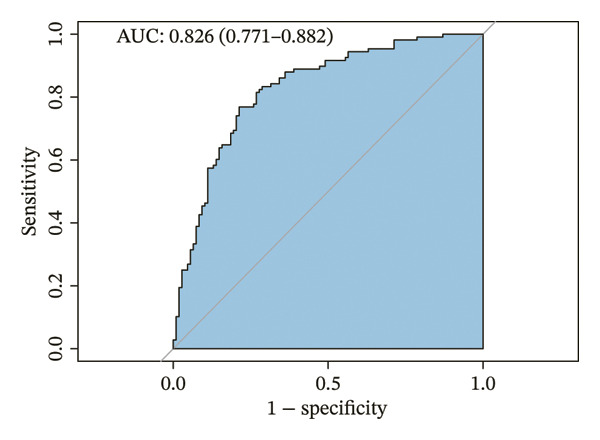
(b)

**Figure:**
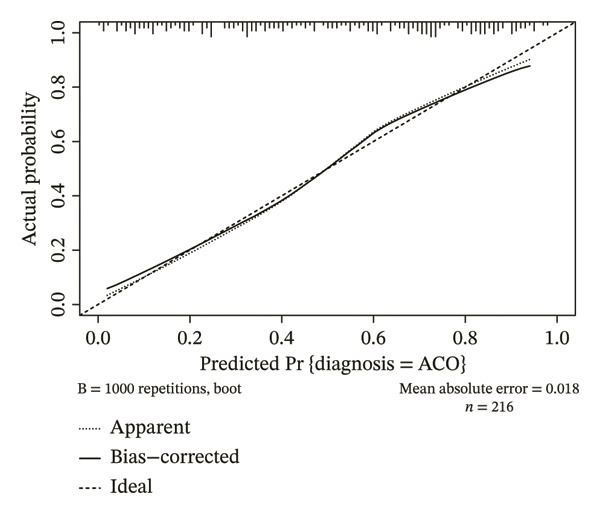
(c)

## 4. Discussion

In this study, we explored the quantitative CT parameters among the ACO, asthma, and COPD groups and established two diagnostic models based on CT parameters for the differential diagnosis of ACO from asthma and COPD groups. In the first part, we established a CT‐based diagnostic model to distinguish patients with ACO from patients with COPD. The constructed prediction model included sex, age, WA^1st^/BSA, and WT^1st^/BSA, which were determined by univariate and multivariate logistic regression analysis. In the second part, our results show that a combination of sex, age, inferior lobes MD, inferior lobes WA^2nd^%, and superior lobes WA^2nd^% might be prospective parameters for the early diagnosis of ACO between the asthma and ACO groups. The models have promising discrimination properties with AUCs of 0.736 and 0.826, respectively, with great consistency between the predicted and actual probabilities. To our knowledge, this is the first study to investigate the CT features of airway remodeling among asthma, COPD, and ACO patients and build up noninvasive and easily reproducible predictive diagnostic models for diagnosing ACO. The intuitive nomograms have the potential to assist clinicians in decision‐making in clinical practices.

We demonstrated that patients with ACO were older, had lower BMI, and had a higher male ratio than patients with asthma alone. The results were reversed when comparing the ACO group with the COPD groups, which were consistent with other observational studies [28, 29]. We identified imaging differences among the three groups. Our present study showed that the patients in the ACO group had intermediate values of both lungs’ MD and WT^1st^/BSA between the COPD and asthma groups, indicating that the change in structural abnormalities in the airway in patients with ACO was more prominent than in the asthma group and less severe than the COPD group in our study population. This can also explain the intermediate FEV1% predicted values in the ACO group, which were consistent with the previous report [30]. Previous investigations have delved into the pathological distinctions between ACO and the isolated occurrence of COPD or asthma. Individuals with asthma who partake in smoking exhibit an increased presence of goblet cells, mucus‐positive epithelium, and a denser airway epithelium as evidenced by biopsy, compared to nonsmokers [31]. This augmented epithelium is associated with respiratory symptoms, including enhanced phlegm production and dyspnea. In comparison to individuals with COPD, asthmatics with persistent airflow obstruction display a superior CD4+/CD8+ ratio of T cells infiltrating the airway mucosa, diminished emphysema as indicated by CT scan, and a denser reticular basement membrane as observed on bronchial biopsy [32]. Physiologically, this is associated with increased resistance and reactance during both inspiration and expiration, implying that ACO may present a more marked small airways disease compared to isolated asthma or COPD, potentially leading to a more severe clinical outcome [33]. Our findings suggest that the radiographic attributes of denser airways are in alignment with histologic modifications in the airways.

We demonstrated that patients with ACO had a higher ER and thicker airways at the level of the lobar bronchus than subjects with asthma alone, which extended the finding of the prior study [20]. Although the differences in lobar bronchus parameters between patients with features of ACO and those with COPD were not statistically significant in our present study, the patients with ACO indeed had the highest median value among the three groups. These findings suggested that quantitative CT parameters were promising imaging biomarkers for distinguishing ACO from COPD and asthma.

The clinical implications of our research are noteworthy. Given the limitations of lung function tests due to hardware restrictions and insufficient sensitivity, it becomes imperative to explore supplementary diagnostic avenues for a comprehensive evaluation of ACO, especially in situations where spirometry is unavailable. Our study identified the CT features in the ACO group, which were important biomarkers for the early detection of such patients and managing effective treatment plans. In general, previous studies had already drawn attention to the plasma biomarkers of ACO. Previous studies [30, 34] demonstrated that the biomarker profiles associated with inflammation are different between COPD, asthma, and ACO. Plasma neutrophil gelatinase‐associated lipocalin (NGAL) [30] is a promising, valuable biomarker to distinguish patients between ACO and asthma (AUC = 0.7517), while Plasma YKL‐40 [12] may help in the differential diagnosis between ACO and COPD patients (AUC = 0.7138). Jing wang [35] stated that IL‐8 and VEGFA are potential biomarkers to distinguish patients between ACO and asthma, with AUC values of 0.676 and 0.649, respectively. Nevertheless, existing plasma biomarkers still lack the diagnostic accuracy required for clinical ACO identification, thus highlighting the necessity of alternative diagnostic modalities such as CT imaging. Our CT‐based diagnostic models achieved favorable AUC values of 0.736 and 0.826 for ACO diagnosis, outperforming the aforementioned plasma biomarkers (AUC range: 0.649–0.7517). CT imaging and plasma biomarkers each have distinct advantages and inherent limitations: CT enables direct visualization of the characteristic structural lung alterations in ACO to facilitate accurate differential diagnosis, yet it involves ionizing radiation exposure and relatively higher examination costs; plasma biomarkers feature minimal invasiveness and rapid detection for convenient preliminary clinical screening, but they fail to reflect the local structural pathological changes of the lung and lack sufficient diagnostic accuracy for ACO. Collectively, these characteristics underscore the clinical value of their complementary application to improve the diagnostic reliability of ACO, which also represents a key direction for future research in this field.

There are some limitations. First, acknowledging the retrospective nature of the study is important, along with the possibility of inherent selection biases. Moreover, the study was confined to a single institution without external validation, which restricts the generalizability of our diagnostic models. Future research should adopt a prospective, multicenter design to recruit larger, heterogeneous cohorts, with strict harmonization of CT scanning and interpretation protocols to reduce intercenter variability. Additionally, subsequent studies are encouraged to compare the performance of CT‐based models with serum biomarkers and further develop combined models integrating both modalities to enhance diagnostic accuracy and clinical applicability. Second, heterogeneous ACO definitions across studies may affect the generalizability of our findings, and future studies should validate results with standardized criteria. Third, our CT assessment of airway remodeling was limited to the first‐ to third‐generation airways due to software constraints, which prevent the reliable segmentation and quantitative analysis of ultrafine airways beyond this level. Future studies should assess small airway lesions below the third generation by leveraging emerging technologies, including ultrahigh‐resolution CT and advanced postprocessing tools. Fourth, to ensure an adequate sample size, full standardization of CT scanners and slice thicknesses was not feasible. However, we strove to maintain balanced parameter distribution across groups and adopted a unified scanning protocol for all examinations to minimize potential bias from interscanner variability, and we will adopt stricter standardization in future research. Fifth, CT radiation exposure and associated costs remain nonnegligible concerns. These challenges highlight the need for optimized low‐dose CT protocols, cost‐effective implementation strategies, and standardized training to mitigate such limitations for broader clinical adoption.

## 5. Conclusions

In summary, we report promising noninvasive CT‐based approaches for the differential diagnosis of ACO from asthma and COPD in clinical practice. This study highlights the potential of quantitative CT methods for evaluating airway remodeling features. We expect that our models can be easily extended to clinical studies, therefore improving management and guiding treatment for specific patients.

**Nomenclature**
ACO: Asthma–chronic obstructive pulmonary disease overlap
AUC: Area under the curve
BMI: Body mass index
BSA: Body surface area
COPD: Chronic obstructive pulmonary disease
CI: Confidence interval
ER: Emphysema ratio
FEV1: Forced expiratory volume in 1 second
FVC: Forced vital capacity
GINA: Global Initiative for Asthma
GOLD: Global Initiative for Chronic Obstructive Lung Disease
ROC: Receiver operating characteristic
SD: Standard deviation
WT: Wall thickness
WA: Wall area
WA%: Wall area percentage

## Author Contributions

Mei‐Cheng Chen: data curation, formal analysis, investigation, writing–original draft, writing–review and editing, and visualization.

Yang‐Li Liu: data curation, formal analysis, investigation, and writing–review and editing.

Feng‐Jia Chen: data curation, formal analysis, and investigation.

Wei Liang: data curation, formal analysis, and investigation.

Yu‐Biao Guo: data curation, formal analysis, and investigation.

Wei‐Wei Deng: data curation, formal analysis, and investigation.

Ling Ma: data curation, formal analysis, and investigation.

Ying Zhu: conceptualization, methodology, investigation, project administration, supervision, writing–review and editing, and validation.

Shi‐Ting Feng: conceptualization, methodology, investigation, project administration, supervision, writing–review and editing.

## Funding

This study was supported by the National Natural Science Foundation of China (82100054), GuangDong Basic and Applied Basic Research Foundation (2020A1515110933), and the Science and Technology Project of Guangzhou City (202102020078).

## Disclosure

All authors provided consent for publication.

## Ethics Statement

This study was approved by the Institutional Ethics Committee of the First Affiliated Hospital of Sun Yat‐sen University (No. [2021]072). Informed consent was waived. The authors are accountable for all aspects of the work in ensuring that questions related to the accuracy or integrity of any part of the work are appropriately investigated and resolved.

## Consent

Please see the Ethics Statement.

## Conflicts of Interest

The authors declare no conflicts of interest.

## Supporting Information

Additional supporting information can be found online in the Supporting Information section.

## Supporting information


**Supporting Information** Additional file 1: Detailed measurement procedures of CT parameters and pulmonary function test.

## Data Availability

The datasets generated and analyzed during the current study are not publicly available due to containing sensitive patient information but are available from the corresponding author upon reasonable request.
