# Supplementary material for: Computed Tomography–Based Evaluation of Airway Remodeling for Distinguishing Asthma–Chronic Obstructive Pulmonary Disease Overlap From Asthma and Chronic Obstructive Pulmonary Disease
Source: Can Respir J. 2026 May 30;2026:4348540. doi: 10.1155/carj/4348540 (PMC13239247; doi:10.1155/carj/4348540)
Supplement: Supplementary file 1 — Supporting Information Additional file 1: Detailed measurement procedures of CT parameters and pulmonary function test. [file CARJ-2026-4348540-s001.zip › FigureS1.pdf]

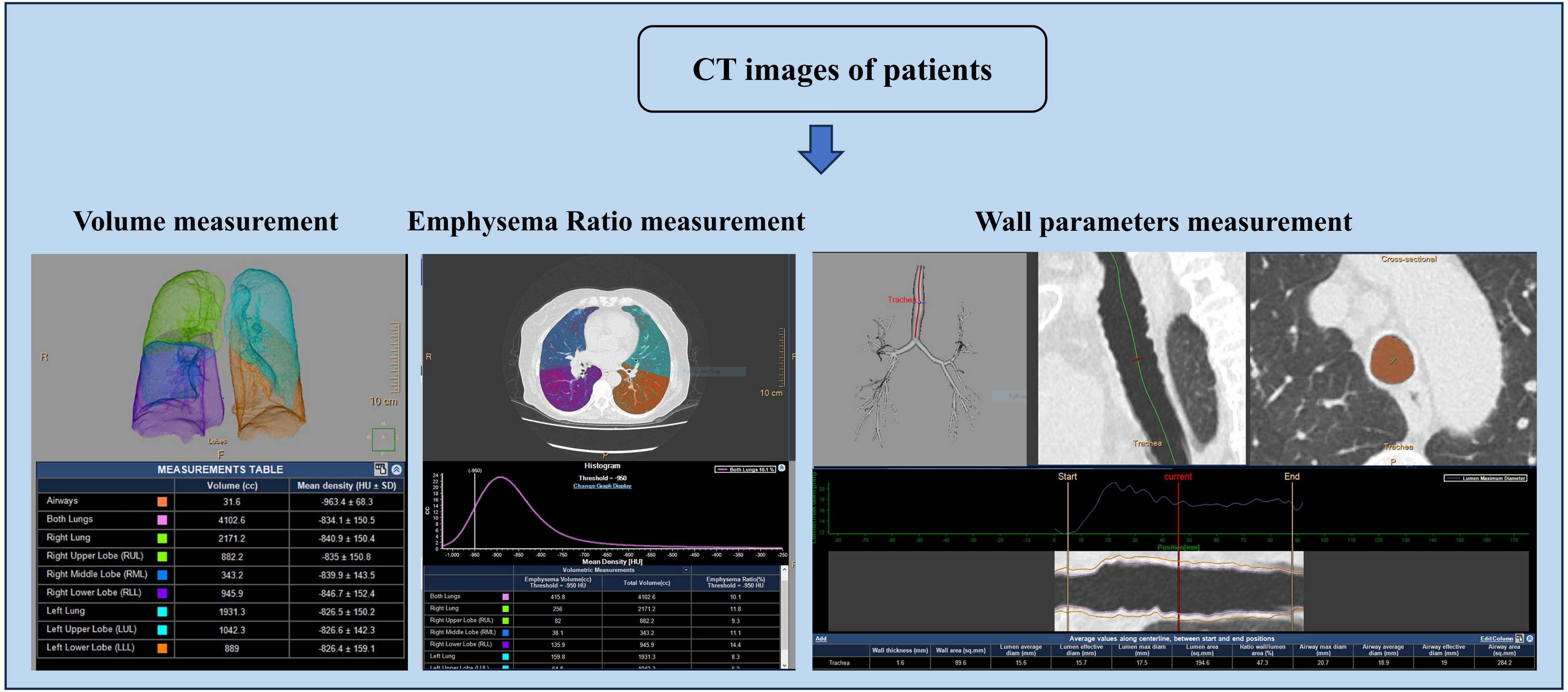
CT images of patients
Volume measurement
Emphysema Ratio measurement
Wall parameters measurement
MEASUREMENTS TABLE
Volume (cc)
Mean density (HU ± SD)
Airways 31.6 -963.4 ± 68.3
Both Lungs 4102.6 -834.1 ± 150.5
Right Lung 2171.2 -840.9 ± 150.4
Right Upper Lobe (RUL) 882.2 -835 ± 150.8
Right Middle Lobe (RML) 343.2 -839.9 ± 143.5
Right Lower Lobe (RLL) 945.9 -846.7 ± 152.4
Left Lung 1931.3 -826.5 ± 150.2
Left Upper Lobe (LUL) 1042.3 -826.6 ± 142.3
Left Lower Lobe (LLL) 889 -826.4 ± 159.1
10 cm
Histogram
Threshold = -950
Mean Density [HU]
Start
current
End
Trachea
Cross-sectional
