# Supplementary material for: Computed Tomography–Based Evaluation of Airway Remodeling for Distinguishing Asthma–Chronic Obstructive Pulmonary Disease Overlap From Asthma and Chronic Obstructive Pulmonary Disease
Source: Can Respir J. 2026 May 30;2026:4348540. doi: 10.1155/carj/4348540 (PMC13239247; doi:10.1155/carj/4348540)

****Supporting Information****

Methods

Measurement of quantitative CT parameters

CT parameters analysis was performed with image-analyzing software (VIDA Apollo Version 2.0) for the measurement of quantitative CT parameters. Firstly, the original images of the inspiratory phase are imported into the software to automatically distinguish the lung tissue from the adjacent structures and identify the lung fissure and lobes. If there is a deviation in partial segmentation, a manual auxiliary correction would be carried out to correct the results (Figures S1). The analysis was blinded to patients’ information.

In each generation, a measured point was selected in the middle of each bronchus. We measured lung density parameters: ER (Emphysema ratio of the lung, the % area of low attenuate(<-950HU) to the corresponding lung area), MD (Mean density of the lung). And we also measured bronchial parameters of 1^st^-3^rd^ generation airways: wall thickness (WT, the mean distance from the outer edge to the inner edge of the airway, mm), wall area (WA, the area of the airway wall, sq. mm) and wall area percentage (WA%, calculated by WA/ the area of the airway). To simplify the calculation, we defined the trachea and main bronchus as the first level airway, which was calculated by the mean value of airway and main bronchus parameters. And defined the segmental bronchus as the second-level airway: Superior lobes parameters were calculated by the mean value of parameters of the upper lobes of both lungs and the middle lobe of the right lung; Inferior lobes parameters were calculated by the mean value of parameters of lower lobes of both lungs; Because the bronchial parameters were correlated to the body size, so we calculated the WT/BSA, WA/BSA to balance the effect.

Measurement of Pulmonary Function Test

Lung function test results were obtained by a pneumotachograph-based systemin according to the recommendations of the European Respiratory Society. Briefly, we measured the following parameters: the FEV1(forced expiratory volume in 1 second), FEV1% predicted (Calculated according to the previously determined reference value of healthy Chinese crowd), and FVC (forced vital capacity).

Results

**Supplementary Figure 1**

The measuring procedures, including Segmentation and volume measurement, Emphysema ratio measurement, and Airways parameters measurement.


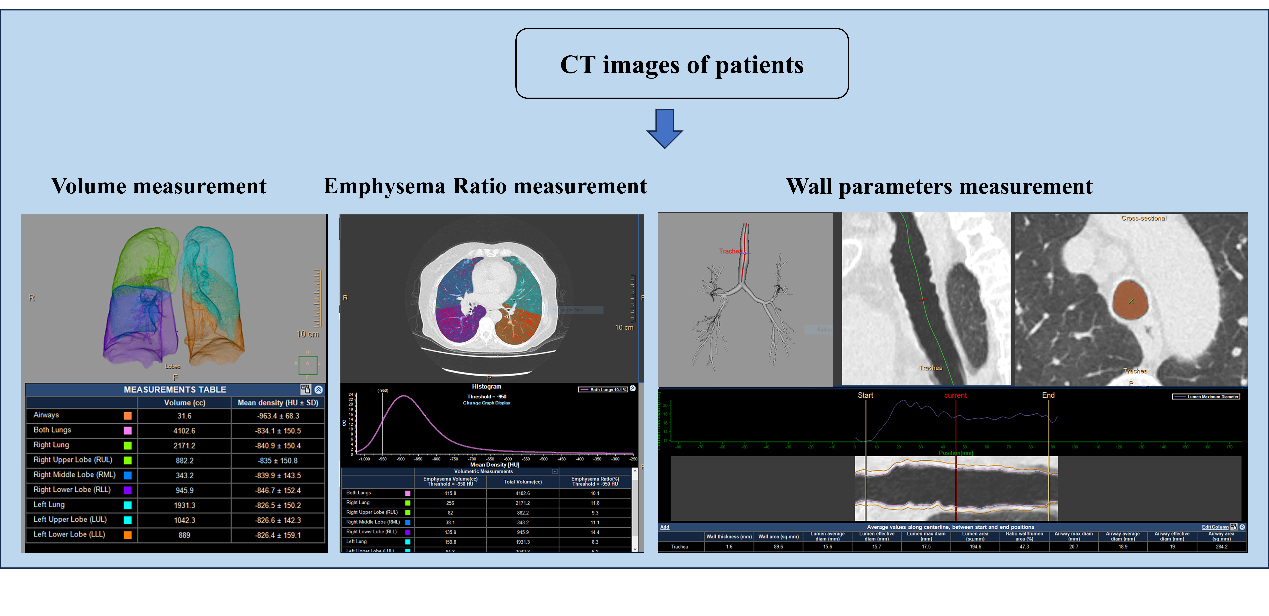

Supplement: Supplementary file 1 — Supporting Information Additional file 1: Detailed measurement procedures of CT parameters and pulmonary function test. [file CARJ-2026-4348540-s001.zip › Supplementary material.docx]
